# Exploring the Structural, Optical, and electrochemical behavior of semicarbazone pyranoquinoline ligand (PQMHC) and its Cu(II) complex for efficient hydrogen peroxide sensing

**DOI:** 10.1038/s41598-025-29948-6

**Published:** 2025-12-11

**Authors:** A. A. El-Saady, A. A.M. Farag, Magdy A. Ibrahim, A. M. Mansour, M. M. El-Nahass, Nesma Salah, Hend S. Magar

**Affiliations:** 1 Thin Film Laboratory, Physics Department, Faculty of Education, Ain Shams University, Roxy, 11757 Cairo Egypt; 2 Department of Chemistry, Faculty of Education, Ain Shams University, Roxy, 11757 Cairo Egypt; 3 Solid State Physics Department, Physics Research Institute, National Research Centre, Dokki, 12622 Giza Egypt; 4 Applied Organic Chemistry Department, National Research Centre (NRC), 33 El Bohouth St, Dokki, 12622 Giza Egypt

**Keywords:** Pyrano[3,2-c] quinoline, Copper complex, Optical properties, Electrochemical sensing, Hydrogen peroxide, Chemistry, Materials science

## Abstract

The structural, morphological, and optical features of a newly synthesized PQMHC ligand and its Cu(II)-coordinated complex were thoroughly investigated using powder X-ray diffraction (XRD), field emission scanning electron microscopy (FE-SEM), and UV–Vis diffuse reflectance spectroscopy. XRD results confirmed that the PQMHC ligand crystallizes in an orthorhombic phase (space group *Imma*), while the Cu(II)-PQMHC complex exhibits a monoclinic phase (space group *P2₁/m*), indicating successful coordination with the metal center. FE-SEM images showed vertically aligned nanofibers, with average diameters of approximately 73 nm for the ligand and 52 nm for the complex, supporting their potential in optoelectronic applications. Diffuse-reflectance UV-Vis spectroscopy coupled with Kubelka–Munk/Tauc analysis yielded optical band gaps of (2.661 and 2.460 eV in the direct transition case) and (2.305 and 1.896 eV in the indirect transition case) for PQMHC and Cu(II)–PQMHC, respectively, consistent with charge-transfer-mediated gap narrowing upon complexation. The electrochemical properties of the PQMHC ligand and its Cu(II)-PQMHC complex were investigated using cyclic voltammetry (CV) and electrochemical impedance spectroscopy (EIS). Both the ligand and its Cu(II) complex demonstrated efficient electron transfer and rapid, linear sensitivity toward hydrogen peroxide detection within the 0.05–1000 µM range and a detection limit of 0.009 µM using the chronoamperometric (CA) technique. These enhanced electrochemical characteristics suggest their potential suitability for applications in sensors and biosensors.

## Introduction

Organic semiconductors featuring π-conjugated frameworks and delocalized π-electrons exhibit a range of desirable biological, electrical, and optical properties, rendering them highly suitable for integration into optoelectronic devices^1,2^. Among these, nitrogen-containing heterocycles such as quinolines are of particular interest due to their structural versatility and functional potential. The inherent advantages of organic materials, including high surface area, cost-effectiveness, and mechanical flexibility, have led to their widespread application in various fields such as sensors, photovoltaic systems, conductive components, imaging technologies, and other optoelectronic platforms^3–5^.

Quinoline Schiff bases (QSBs) and their transition metal complexes, particularly those incorporating Co(II), Ni(II), and Cu(II) ions, have attracted considerable attention due to their promising antimicrobial, antibacterial, and antifungal activities, as well as their potential applications in various pharmacological fields^6–9^.

In recent years, coordination compounds based on transition metals, particularly copper(II), have attracted significant attention in electrochemical sensing applications owing to their favorable redox properties and catalytic activity toward H₂O₂ reduction or oxidation^10^. The design of efficient ligands capable of stabilizing metal centers while enhancing electron transfer kinetics is pivotal in developing high-performance sensors. Semicarbazone derivatives, known for their versatile coordination behavior and biological relevance, have been increasingly explored as ligands in metal complex synthesis^11^. When integrated into heterocyclic frameworks such as pyrano[3,2-c]quinoline, these ligands can offer extended π-conjugation, improved stability, and tunable electronic properties—features beneficial for both optical and electrochemical applications^12–15^.

Semicarbazone ligands, characterized by their azomethine and carbonyl donor sites, are widely studied for their robust coordination chemistry and tunable electronic properties when complexed with transition metals such as Cu(II)^16,17^. These coordination complexes adopt predictable geometries—often square-planar or distorted octahedral—and exhibit distinctive spectroscopic features in both IR and UV–vis regions, which reflect ligand-to-metal (LMCT) and intra-ligand (ILCT) transitions^18^.

In particular, metal complexes derived from semicarbazones fused with heterocyclic aromatic systems (e.g., quinoline-based frameworks) demonstrate enhanced photophysical and electrochemical behavior. Recent studies of copper(II) complexes with heterocyclic semicarbazones report significant UV–vis absorption shifts, fluorescence quenching, and characteristic coordination bands in IR spectra (e.g. ν(C = N), ν(Cu–N), ν(Cu–O))—markers consistent with formation of stable Cu–N and Cu–O bonds^16^.

Furthermore, theoretical methods such as density functional theory (DFT) and Hirshfeld surface analysis have been employed to interpret structural stability, frontier molecular orbitals (HOMO–LUMO), and intermolecular interactions in such complexes^18^. These techniques support experimental observations and provide insight into the charge transfer behavior intrinsic to ligand–metal systems.

Pyrano[3,2-c]quinoline cores are no strangers to optoelectronic scrutiny; recent TD-DFT and photovoltaic studies have highlighted their exceptional π-delocalisation and charge-transfer propensities^19,20^. Yet the deliberate grafting of a semicarbazone appendage to this chromophore—and the subsequent chelation of Cu(II)—remains conspicuously absent from the sensing literature. This is surprising. Semicarbazones, particularly those derived from pyridoxal or heteroaromatic aldehydes, are well documented to stabilise Cu(II) centres in geometries that facilitate rapid Cu(II)/Cu(I) cycling, a prerequisite for low-potential H₂O₂ electro-reduction^21–23^. Moreover, the electron-rich pyranoquinoline platform should intensify ligand-to-metal charge transfer (LMCT) bands, offering an internal ratiometric optical handle that bypasses the fluorescence intermittency plaguing quantum-dot reporters^21–23^.

The accurate determination of hydrogen peroxide is of significant importance across various fields, including environmental monitoring, clinical diagnostics, pharmaceuticals, food safety, household cleaning products, and fuel cells^24^. Additionally, monitoring changes in H_2_O_2_ concentration is critical in biomedical research, particularly in detecting drug-induced apoptosis in cancer cells, which can aid in chemically targeting and inhibiting cancer progression.

Given this wide range of applications, there is a growing demand for sensitive, cost-effective, and reliable sensors for H_2_O_2_ detection. Numerous analytical techniques have been employed, such as electrochemical methods^25^, chemiluminescence^26^, fluorescence^27^, and spectrophotometry^28^. Among these, electrochemical detection stands out due to its simplicity, high sensitivity, rapid response, and strong selectivity, making it the most promising approach for practical applications^29^. The electrochemical performance of a sensor is primarily governed by the nature of the materials incorporated onto the electrode surface, which may be nanoparticles^30^, nanoporous^31^, organic compounds^32^ and, in particular, organometallic systems-most notably those based on copper-which have attracted considerable attention^33^. Organometallic compounds, in particular, have emerged as highly promising redox mediators^34^. Therefore, the development of novel nanomaterials with superior electrocatalytic activity is essential for enhancing the sensitivity and overall performance of electrochemical sensors.

Based on this background, we introduce a novel ligand combining a pyranoquinoline core with a semicarbazone moiety—denoted PQMHC—designed to provide strong metal chelation and favorable optical-electrochemical characteristics. Complexation with Cu(II) is expected to yield a molecule with pronounced spectroscopic signatures (UV–vis absorption) and defined redox activity suitable for electrochemical study.

This manuscript reports the synthesis and characterization of PQMHC and its Cu(II) complex through XRD, FE-SEM, UV–vis spectroscopy, and electrochemical behavior, which is investigated via cyclic voltammetry and related techniques to elucidate the redox properties of the Cu(II) center coordinated to PQMHC. Our comprehensive approach aims to correlate structural features with optical and electrochemical responses, thus demonstrating the utility of the Cu(II)–PQMHC system as a model platform for functional ligand-metal assemblies.

## Experimental

### Materials

The chemical CuSO_4_.5H_2_O was employed as BDH. Disodium ethylenediaminetetraacetic acid (EDTA) salt, nitric acid, ammonium hydroxide, and murexide were obtained from either BDH or Merck. Reagent-grade organic solvents (ethanol, diethyl ether, dimethylformamide [DMF]) were employed throughout the study without additional purification. Hydrogen peroxide (H_2_O_2_), potassium ferricyanide, potassium ferrocyanide, potassium dihydrogen phosphate, potassium mono-hydrogen phosphate, potassium chloride (KCl), hydrochloric acid (HCl), and sodium hydroxide (NaOH) were provided by Sigma-Aldrich.

### **Synthesis of the H**_**2**_**L ligand**

**Fig. 1:**
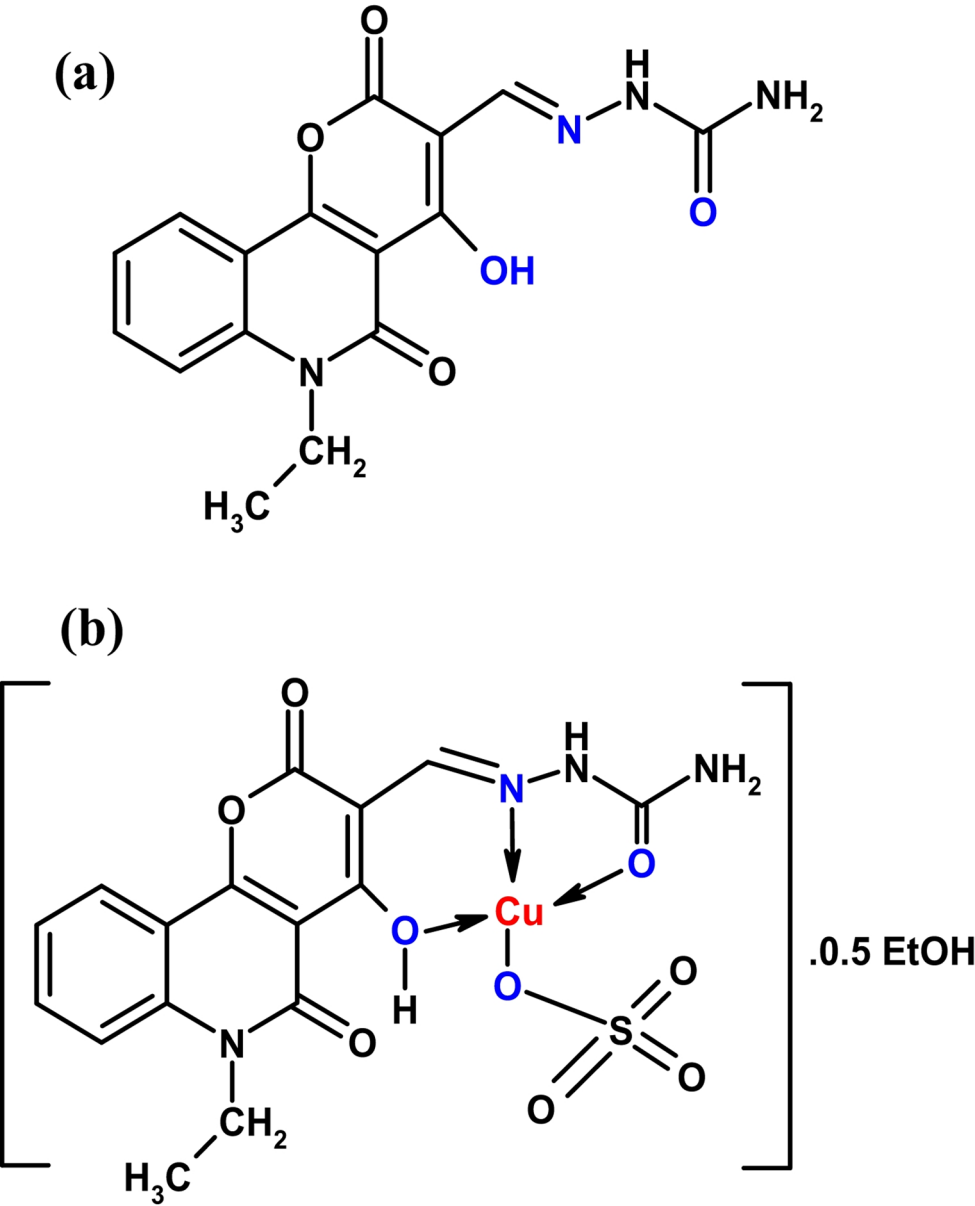
Molecular structures of (a) **PQMHC** and (b) the Cu(II)-**PQMHC** complex.

The 2-[(6-ethyl-4-hydroxy-2,5-dioxo-5,6-dihydro-2 H-pyrano[3,2-c]quinolin-3-yl)methylidene] hydrazinecarboxamide (PQMHC) ligand (Fig. 1a) was synthesized following an established procedure reported in the literature^14^. This hydrazone-based ligand was derived from the condensation of 6-ethyl-4-hydroxy-2,5-dioxo-5,6-dihydro-2 H-pyrano[3,2-c]quinoline-3-carbaldehyde with semicarbazide hydrochloride under controlled reaction conditions. The resulting hydrazone product was purified through recrystallization from acetic acid, yielding a high-purity compound suitable for further coordination studies. The structural integrity of the synthesized PQMHC ligand was confirmed using spectroscopic techniques, including FT-IR, NMR, and elemental analysis, as documented in prior work^14^. Its distinctive pyrano-quinoline scaffold, coupled with a semicarbazide functional group, renders it an effective chelating agent for transition metal ions, particularly Cu(II), due to its multidentate binding sites.

### Synthesis of the Cu(II)-PQMHC complex

An aqueous solution of LiOH·H_2_O (0.08 g, 2.00 mmol in 5 mL water) was added dropwise with stirring maintained throughout to a hot solution of the H_2_L ligand (0.52 g, 2.00 mmol). LiOH·H₂O was added to promote deprotonation of the acidic hydrogen and generate a possible coordinating anion. However, experimental results confirmed that it did not participate in the complex formation. CuSO_4_.5H_2_O (0.59 g, 2.00 mmol in 20 mL ethanol) was gradually added under continuous stirring, providing a 1:1 molar ratio. Refluxing the reaction mixture for 6 h led to the formation of a yellow solid. The solid was then filtered, washed with ethanol and diethyl ether, and finally air-dried. The obtained yield (Fig. 1b) was 0.76 g (72%). This proposed structure was confirmed using spectroscopic methods as discussed before.

### Characterization techniques

The crystal structures of the PQMHC ligand and its Cu(II) complex were investigated using a Bruker D8 Advance X-ray diffractometer equipped with Cu K_α_ radiation (λ = 1.54059 Å). The instrument operated at 40 kV and 40 mA, with diffraction patterns collected over a 2θ range of 5° to 70° at a step size of 0.05°. The resulting data were analyzed using CRYSFIRE 2020 software version 1.0.5 [http://ccp14.cryst.bbk.ac.uk/Crysfire.html] for indexing and CHECKCELL (version 3, 2000) [http://ccp14.cryst.bbk.ac.uk/tutorial/lmgp/chekcellb.htm] for refinement to determine lattice parameters, crystallite size, and microstrain.

The surface morphology and nanostructural features of the samples were examined using a Quanta FEG 250 field emission scanning electron microscope (FE-SEM). The measurements were conducted at an accelerating voltage of 20 kV to ensure high-resolution imaging. The obtained micrographs were processed using ImageJ software version 1.54 g [https://imagej.net/ij/download.html] to determine particle size distribution and average nanofiber dimensions.

The optical absorption properties of the synthesized compounds were analyzed using a Jasco V-570 spectrophotometer with an integrating sphere attachment for diffuse reflectance measurements. Spectra were recorded at room temperature in the wavelength range of 200–2500 nm. The Kubelka-Munk function was applied to convert reflectance data into absorption spectra, and Tauc plots were used to estimate the direct and indirect band gaps. Empirical models were subsequently used to calculate refractive indices and dielectric constants.

### Electrochemical measurements

A screen-printed electrode (SPE) platform was employed to assess the electrochemical behavior of the synthesized nano compounds via cyclic voltammetry (CV) utilizing a CHI-potentiostat. SPEs were chosen due to their low power requirements, high sensitivity, fast and linear response, and reliable performance at ambient conditions, making them an ideal platform for in-situ electrochemical sensing.

Further electrochemical investigations were conducted using a CHI potentiostat to perform electrochemical impedance spectroscopy (EIS). In all experiments, either unmodified (bare) or nano-compound-modified SPEs served as the working electrode, while integrated carbon and silver disks functioned as the counter and reference electrodes, respectively.

Electrochemical analyses were carried out in triplicate using a 5 mM equimolar solution of [Fe(CN)₆]³⁻/⁴⁻ in 0.1 M KCl as the supporting electrolyte. CV measurements were recorded over a potential range from − 1.0 V to + 1.0 V at a scan rate of 50 mV/s. EIS measurements were performed at open circuit potential using a 10 mV AC perturbation across a frequency range of 10,000 Hz to 0.1 Hz. The resulting Nyquist plots were fitted with an equivalent electrical circuit to extract parameters such as charge transfer resistance (R_ct_).

Chronoamperometric measurements were also conducted in phosphate-buffered saline (PBS) at a fixed potential of 0.7 V to evaluate the sensor’s steady-state response. All electrochemical experiments were performed at room temperature (25 ± 2 °C).

To modify the electrode surface, 5 mg of the synthesized nano compounds were dispersed in 1.0 mL of double-distilled water and sonicated for 30 min to ensure uniform dispersion. A 30 µL aliquot of the resulting suspension was drop-cast onto the SPE surface and air-dried at room temperature. Residual or loosely bound materials were removed by rinsing with double-distilled water.

**Fig. 2:**
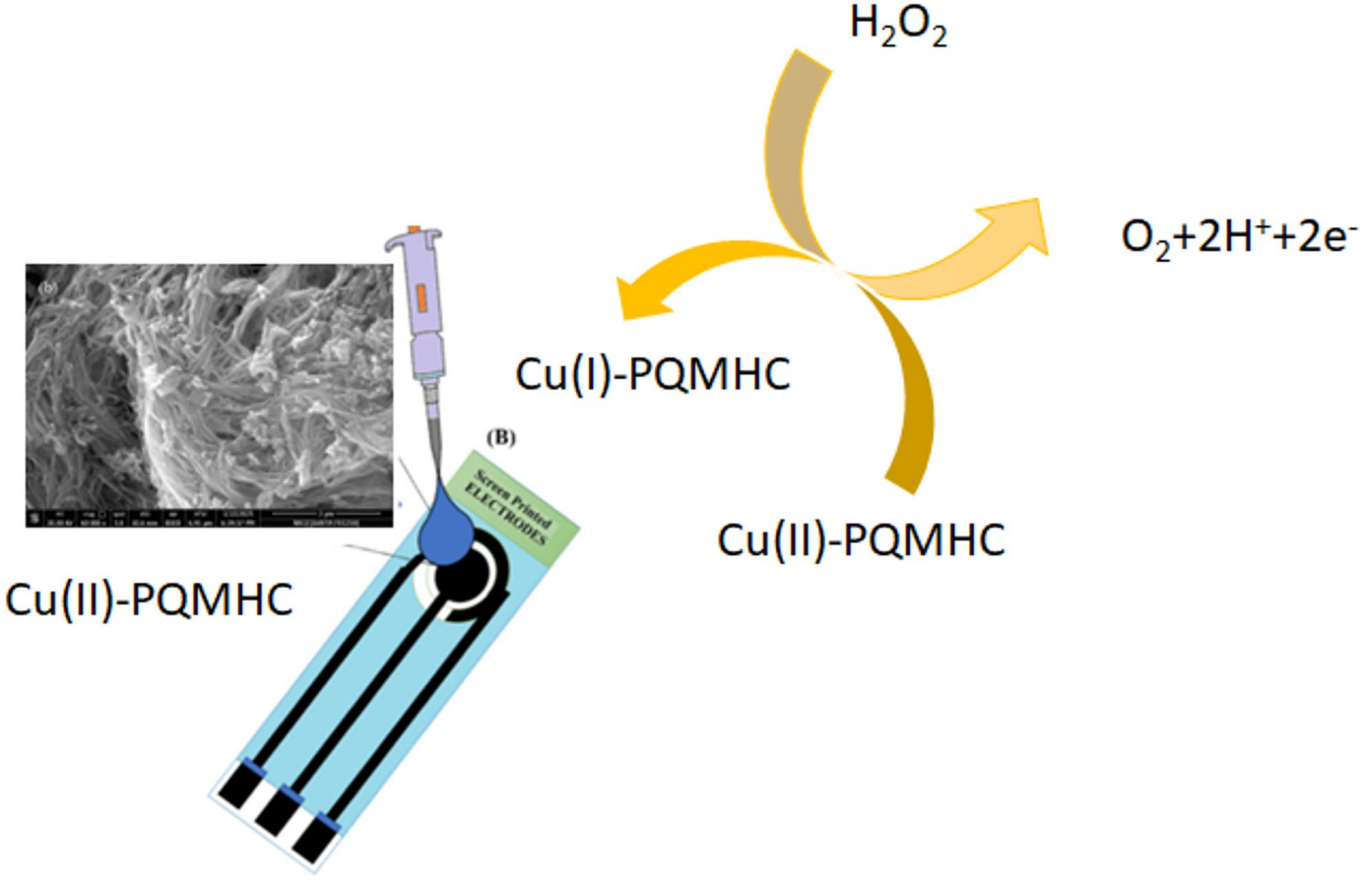
Catalytic mechanism of H₂O₂ reduction/oxidation at the Cu(II)-PQMHC modified Screen-printed electrode (SPE) surface.

The combined electrochemical analyses CV, EIS, and chronoamperometry confirmed the successful surface modification and significantly improved electrochemical properties of the nanocomposite-coated SPEs, as demonstrated in Fig. 2.

## Results and discussion

### XRD evaluation

**Fig. 3:**
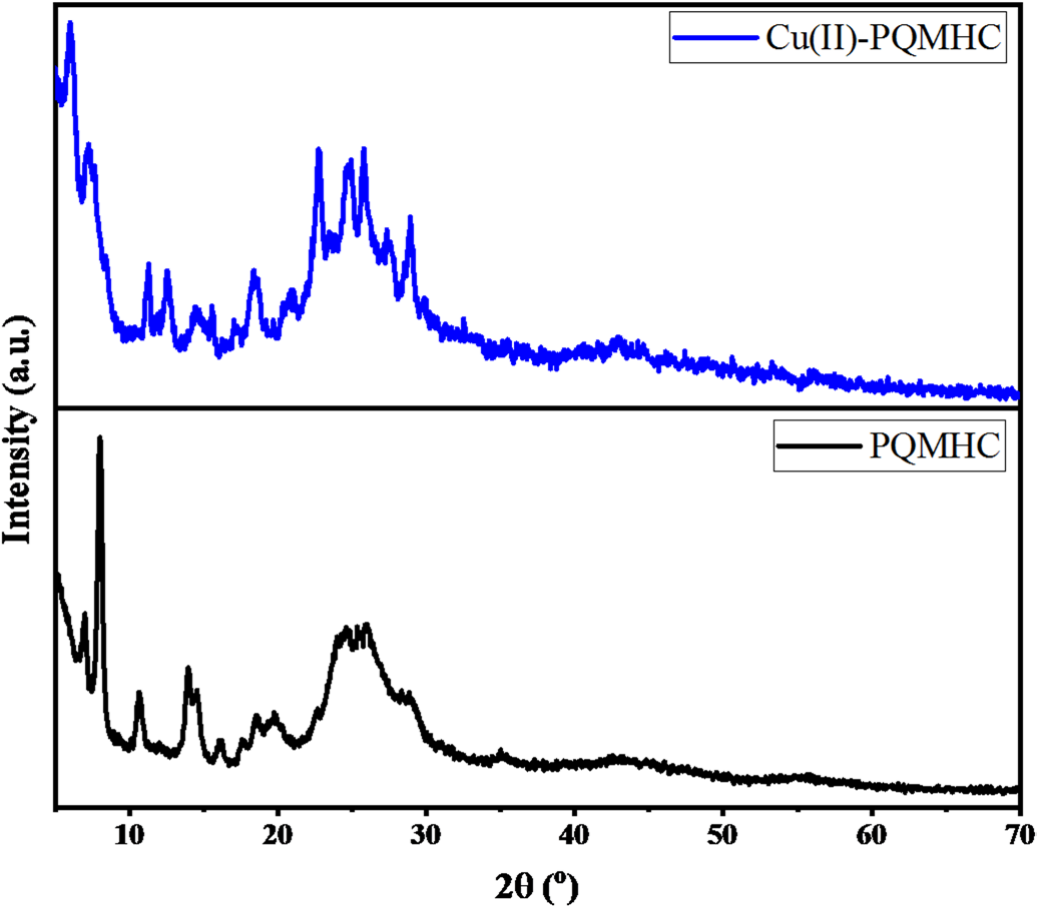
XRD patterns of the **PQMHC** ligand and its Copper (II) complex in the powder form.

Figure 3 presents the XRD patterns of both PQMHC and its copper complex. The diffraction patterns reveal multiple peaks of varying intensities, indicating the polycrystalline nature of both the free ligand and the Cu(II) complex.

The diffraction peaks of both PQMHC and Cu(II)-PQMHC compounds were indexed using theCRYSFIRE 2020 software package version 1.0.5 [http://ccp14.cryst.bbk.ac.uk/Crysfire.html]^35^. The analysis revealed that the PQMHC ligand crystallizes in an orthorhombic system with space group *Imma*, and lattice parameters of *a* = 44.147 Å, *b* = 25.149 Å, *c* = 4.590 Å, with α = β = γ = 90°. The relatively large *a* and *b* values are attributed to the extended π-conjugated aromatic framework of PQMHC, which promotes π–π stacking and hydrogen-bonding interactions among adjacent molecules, resulting in an expanded crystal lattice.

In contrast, the Cu(II)-PQMHC complex adopts a monoclinic crystal system with space group *P2*_*1*_*/m*, and unit cell dimensions of *a* = 17.195 Å, *b* = 15.645 Å, *c* = 12.543 Å, α = γ = 90°, and β = 121.36°. The distinct shift in lattice dimensions and symmetry compared to the free ligand confirms structural reorganization due to metal coordination. Although single-crystal XRD measurements are not yet available, they will be undertaken in future work to further refine and validate the structural parameters obtained from powder data.

Further refinement was conducted to ensure accuracy in indexing and phase assignment using the CHECKCELL software (version 3, 2000).

[http://ccp14.cryst.bbk.ac.uk/tutorial/lmgp/chekcellb.htm]^36^, which enabled the assignment of Miller indices (*hkl*) to each diffraction peak. Table 1 summarizes the observed and calculated 2θ values, interplanar spacings (*d*_*hkl*_), relative intensities (I/I₀), full width at half maximum (FWHM), and assigned Miller indices for both the ligand and its Cu(II) complex. Notable differences in diffraction angles and intensities between the free ligand and the complex confirm the successful coordination of the copper ion with PQMHC, resulting in the formation of a new crystalline phase. These structural insights provide valuable information on the orientation and arrangement of crystal planes, contributing to a deeper understanding of the physicochemical properties and potential functional applications of both compounds.

**Table 1:** XRD indexing data for **PQMHC** ligand and its copper (II) complex in the powder form.

PQMHC											Cu(II)-PQMHC									
Peak no.	2θ (°)		d (Å)		I/Io (%)	FWHM (°)	h	k	l	Peak no.		2θ (°)		d (Å)		I/Io (%)	FWHM (°)	h	k	l
	observed	calculated	observed	calculated								observed	calculated	observed	calculated					
1	6.998	7.024	12.6214	12.5745	52.4	1.04876	0	2	0	1		5.999	6.014	14.7207	14.6830	100	0.89894	1	0	0
2	7.996	8.004	11.0482	11.0367	100	0.4422	4	0	0	2		7.247	7.250	12.1882	12.1833	71.5	1.44829	-1	0	1
3	10.643	10.657	8.3056	8.2948	31.2	0.82263	4	2	0	3		8.346	8.252	10.5856	10.7064	45.2	0.54935	-1	1	0
4	13.939	13.934	6.3482	6.3505	37.8	0.64392	6	2	0	4		11.293	11.302	7.8290	7.8225	43.4	0.61261	0	2	0
5	14.539	14.638	6.0875	6.0467	31.8	0.63469	2	4	0	5		12.491	12.500	7.0806	7.0757	41.8	0.91979	1	0	1
6	16.037	16.048	5.5221	5.5184	18.2	0.14982	8	0	0	6		14.389	14.344	6.1507	6.1698	33.3	0.24971	-1	0	2
7	17.585	17.536	5.0393	5.0532	18.4	0.69918	8	2	0	7		15.538	15.490	5.6983	5.7159	33.5	0.54388	-3	0	1
8	18.534	18.548	4.7834	4.7799	25	0.79906	6	4	0	8		17.036	17.019	5.1406	5.2055	28.9	1.44829	-3	0	2
9	19.733	19.645	4.4954	4.5154	25.8	0.39953	0	1	1	9		18.384	18.445	4.8221	4.8062	41.9	0.54745	-2	2	2
10	22.679	22.674	3.9176	3.9184	26.7	0.69917	4	6	0	10		20.881	20.877	4.2507	4.2515	37.3	0.04994	-2	3	0
11	23.978	23.959	3.7083	3.7112	46.1	0.19976	7	0	1	11		22.280	22.303	3.9869	3.9828	49.3	0.89894	-1	3	2
12	24.577	24.620	3.6192	3.6130	48.7	0.19977	10	4	0	12		22.729	22.717	3.9091	3.9112	70.3	0.79906	0	4	0
13	25.326	25.202	3.5139	3.5309	48.9	0.04994	12	2	0	13		24.727	24.729	3.5976	3.5973	66.7	0.14982	2	3	1
14	26.025	26.048	3.4210	3.4181	49.5	0.34959	5	4	1	14		24.927	24.930	3.5692	3.5688	67.8	0.29965	-3	3	0
15	26.425	26.575	3.3702	3.3515	44.4	0.89894	9	0	1	15		25.776	25.788	3.4535	3.4519	70.4	0.99882	-2	4	0
16	27.424	27.400	3.2496	3.2524	34.4	0.14982	8	3	1	16		26.674	26.660	3.3393	3.3410	48.2	0.14982	-4	2	3
17	28.323	28.278	3.1485	3.1534	31.6	0.14982	14	0	0	17		27.324	27.331	3.2613	3.2605	51.4	0.09988	-2	3	3
18	28.822	28.894	3.0951	3.0876	31.2	0.19976	1	6	1	18		28.522	28.522	3.1270	3.1270	43.7	0.49941	-3	4	2
19	29.671	29.542	3.0084	3.0213	22.9	0.2497	11	0	1	19		28.922	28.919	3.0846	3.0849	54.5	0.79249	-2	0	4
20	30.021	30.015	2.9742	2.9748	20.3	0.14982	10	3	1	20		29.871	29.861	2.9888	2.9897	35.5	0.54935	-4	1	4

**Fig. 4:**
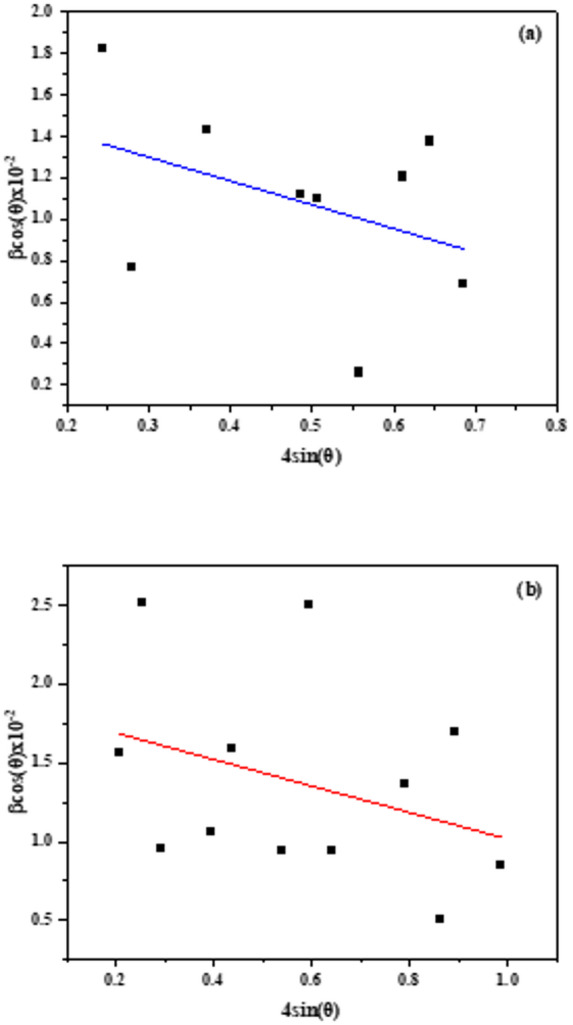
Plot of βcos(θ) vs. 4sin(θ) for (a) **PQMHC** and (b) **Cu(II)-PQMHC** complex.

The average crystallite size and lattice strain of the investigated materials were determined using the Williamson–Hall (W–H) method^37^. Figure 4a and b present the W–H plots of βcos θ versus 4sin θ for PQMHC and Cu(II)-PQMHC, respectively. The calculated average crystallite size and microstrain for the PQMHC ligand were 9.17 nm and 11.52 × 10^− 3^, respectively, while for the Cu(II)-PQMHC complex, values of 8.11 nm and 8.45 × 10^− 3^ were obtained.

### Morphological analysis

The field emission scanning electron microscopy (FE-SEM) images provided offer detailed insight into the surface morphology and microstructural differences between the free ligand Semicarbazone Pyranoquinoline (PQMHC) and its corresponding Cu(II) complex (Cu(II)–PQMHC).

**Fig. 5:**
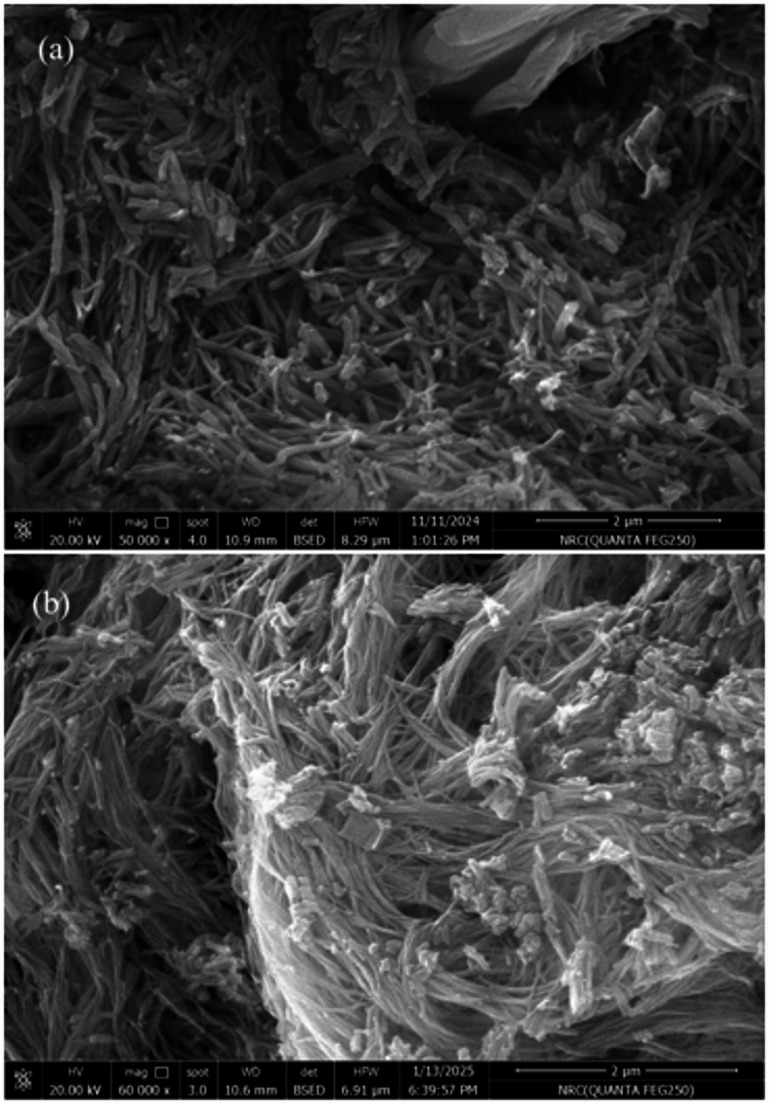
SEM image for the surface morphology of (a) **PQMHC** and (b) **Cu(II)-PQMHC** complex.

In Fig. 5a, the FE-SEM micrograph of the pristine PQMHC ligand reveals a loosely packed, fibrous-like morphology with a high density of elongated, needle-shaped particles. These structures are randomly oriented, forming a somewhat porous and disordered network. Such morphology is indicative of an amorphous or semi-crystalline nature, which is typical for many organic ligands before metal coordination. Fibrous architecture suggests potential for high surface area, which may enhance the ligand’s reactivity and interaction with metal ions.

In Fig. 5b, the SEM image of the Cu(II)–PQMHC complex shows a significant morphological transformation upon coordination with **Cu(II)** ions. The microstructure exhibits a denser, more compact arrangement with thicker bundles and an interwoven network of nanofibers. The individual fibers appear to have decreased in diameter and exhibit more pronounced entanglement, reflecting an enhanced structural order and aggregation likely induced by metal-ligand coordination. This morphological evolution is attributed to the chelation of Cu(II) ions with the donor atoms of the ligand (e.g., nitrogen and oxygen), which facilitates cross-linking and promotes the formation of a more organized supramolecular assembly.

The observed morphological compaction in the metal complex may also indicate reduced porosity and an increase in crystallinity or molecular packing order, which is commonly associated with coordination complex formation. Moreover, this densification could influence the material’s physicochemical properties, such as thermal stability, mechanical integrity, and electronic behavior, which are critical in catalytic, sensing, or antimicrobial applications.

Well-defined nanofiber structures constitute a distinct class of nanomaterials that serve as ideal platforms for studying linearly polarized emission, dimensionally restricted exciton migration, and efficient electronic coupling factors that collectively contribute to superior electrical and optoelectronic performance^38^. These nanofibers exhibit a variety of morphologies and dimensions, and their high surface-to-volume ratios render them suitable for a wide range of applications, including photovoltaics, gas sensing, and nano-optoelectronic components^39^. In the context of sensing applications, the length of the nanofibers plays a critical role in determining both sensitivity and selectivity. Specifically, extended nanofiber lengths may provide an increased number of active sites for interaction with target analytes, thereby improving the overall sensor response.

**Fig. 6:**
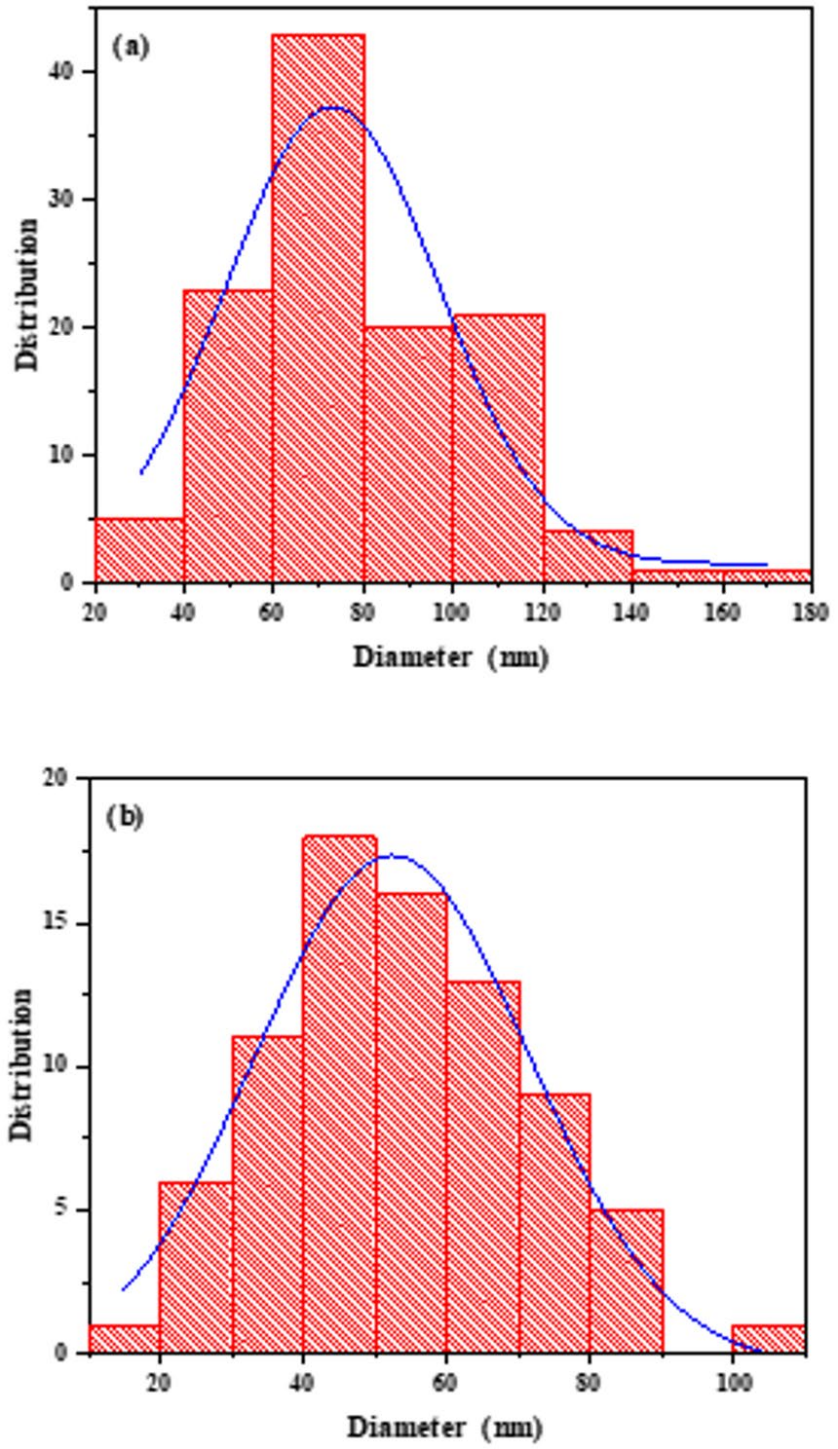
Nanofibers distribution analysis of (a) **PQMHC** and (b) **Cu(II)-PQMHC** complex.

The average nanofiber diameter from the FE-SEM images was determined using the ImageJ image analysis software version 1.54 g [https://imagej.net/ij/download.html]^40^. Statistical evaluations based on the nanofibers’ diameter distribution histograms for the PQMHC ligand and its Cu(II)-PQMHC complex are depicted in Fig. 6a and b, respectively. The PQMHC ligand exhibited nanofiber diameters ranging from 20 to 180 nm, with an average diameter of approximately 73 nm, while the Cu(II)-PQMHC complex showed a narrower distribution between 10 and 110 nm, with a predominant diameter around 52 nm. Notably, the observed nanofiber sizes are larger than the crystallite size derived from X-ray diffraction analysis. This difference arises from the distinct physical scales represented by each technique. The crystallite size obtained from XRD corresponds to the smallest coherently diffracting domains within the crystal lattice, whereas the fiber size observed in SEM images reflects the aggregation of numerous crystallites into larger fibrous structures. Such fibrous morphology likely results from the directional growth and partial coalescence of nanocrystallites during synthesis and solvent evaporation, driven by surface energy minimization rather than random particle agglomeration.

### Optical properties

The optical band gap is a fundamental characteristic of semiconductors and insulators, representing the energy separation between the highest occupied and the lowest unoccupied molecular orbitals^41^. This parameter plays a critical role in defining the electronic and optical behavior of materials. It can be accurately evaluated using techniques such as ultraviolet–visible (UV–Vis) spectroscopy and photoluminescence (PL) spectroscopy. In nanoscale materials, optical absorption is governed not only by discrete electronic transitions but also by quantum confinement effects, which arise due to the reduced dimensionality and particle size^42^. Diffuse reflectance, a phenomenon inherently dependent on particle size, results from the complex interplay of reflection, refraction, diffraction, and absorption of light in multiple directions^43^.

**Fig. 7:**
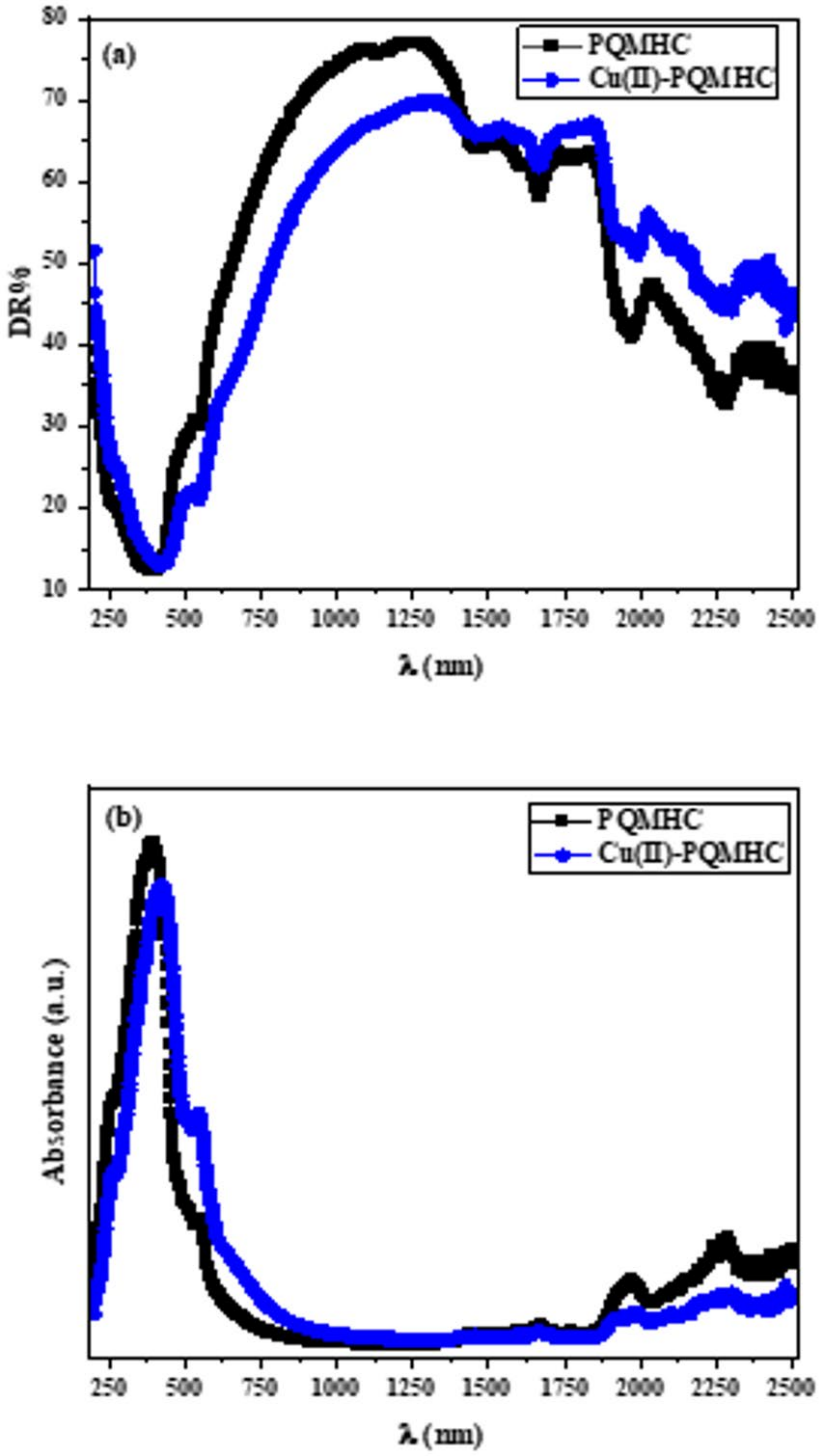
Plot of (a) Diffuse reflectance spectra and (b) Absorptance spectra of **PQMHC** ligand and its Copper (II) complex.

UV–visible diffuse reflectance (DR) spectroscopy was employed to investigate the optical behavior of the PQMHC ligand and its corresponding Cu(II)-PQMHC complex over the spectral range of 200–2500 nm, as illustrated in Fig. 7a. The reflectance spectra of all samples exhibit an initially high reflectance at approximately 190 nm, followed by a pronounced decrease, reaching a minimum near 410 nm. Beyond this point, the diffuse reflectance increases sharply with increasing wavelength, attaining a maximum around 1200 nm for all examined samples. Subsequently, a gradual decline in reflectance is observed with further increases in wavelength. Additionally, interference fringes become apparent in the higher wavelength region. The observed reduction in reflectance at shorter wavelengths, along with the absence of interference fringes, is indicative of the fundamental absorption edge of the films.

The Kubelka–Munk (K–M) function establishes a quantitative relationship between the diffuse reflectance (R) of a material and its corresponding absorbance, expressed as F(R), according to the following equation^44^:

1 $$\:F\left(R\right)\hspace{0.17em}=\hspace{0.17em}{(1\hspace{0.17em}-\hspace{0.17em}R)}^{2}/2R$$

Figure 7b depicts the absorbance spectra of the synthesized PQMHC ligand and its Cu(II)-PQMHC complex. A pronounced absorption band is observed at around 400 nm for the ligand and about 420 nm for the complex.

The optical absorbance of the Semicarbazone Pyranoquinoline ligand (PQMHC) at 400 nm and its Cu(II) complex (Cu(II)–PQMHC) at about 420 nm can be interpreted in terms of electronic transitions and the influence of metal coordination on the ligand’s electronic structure. The shift in absorbance from 400 nm to 420 nm upon complexation with Cu(II) suggests changes in the electronic environment of the ligand, likely due to metal-ligand interactions that alter the energy levels of the molecular orbitals involved in the electronic transitions. The absorbance at 400 nm for PQMHC is indicative of π-π^*^ transitions, which are common in organic compounds with conjugated systems, such as pyranoquinoline structures^45^. Upon coordination with Cu(II), the absorbance shifts to 420 nm, suggesting a change in the electronic structure due to d-d transitions or charge transfer between the metal and the ligand^46,47^.

The coordination of Cu(II) to the ligand can lead to a bathochromic shift (red shift) in the absorbance spectrum, as observed in the shift from 400 nm to 420 nm. This is due to the stabilization of the ligand’s π^*^ orbitals upon complexation, which lowers the energy required for electronic transitions^48,49^. The Cu(II) complex likely involves coordination through nitrogen and oxygen atoms, as seen in similar complexes, which can significantly alter the ligand’s electronic properties^45,49^. While the shift in absorbance provides insights into the electronic changes upon complexation, it is also important to consider other factors such as solvent effects and the specific geometry of the complex, which can further influence the optical properties.

The relationship between a semiconductor’s optical band gap and its absorption coefficient was originally formulated by Wood and Tauc for materials with parabolic electronic bands. In their treatment, the optical band gap *E*_g_​ is obtained from the dependence of the absorption coefficient on the photon energy according to the following expression^50^:

2 $$\:\:F\left(R\right)h\nu\:=\hspace{0.17em}C{\left(h\nu\:-{E}_{g}\right)}^{m}$$

In this equation, hν represents the photon energy, C is a proportionality constant, and Eg​ denotes the optical band gap. The exponent m is a transition-dependent constant that varies according to the nature of the electronic transition: m = 1/2 corresponds to a direct allowed transition, while m = 2 is associated with an indirect allowed transition^51^. The intercept of the extrapolated linear region of the Tauc plot with the energy axis yields the optical band gap of each material.

To elucidate the nature of optical transitions, Tauc plots were constructed for both direct (*m = ½*) and indirect (*m = 2*) allowed transitions. The linearity observed in both plots confirms that the materials can exhibit mixed transition behavior. However, the superior linear fitting and distinct absorption edge in the direct transition plots imply that the dominant electronic process is of the direct allowed type. Such coexistence is common in molecular semiconductors, where localized states and structural disorder can permit weak indirect transitions alongside predominant direct ones. This clarification aligns with the reviewer’s observation and provides a more accurate interpretation of the optical transition behavior.

**Fig. 8:**
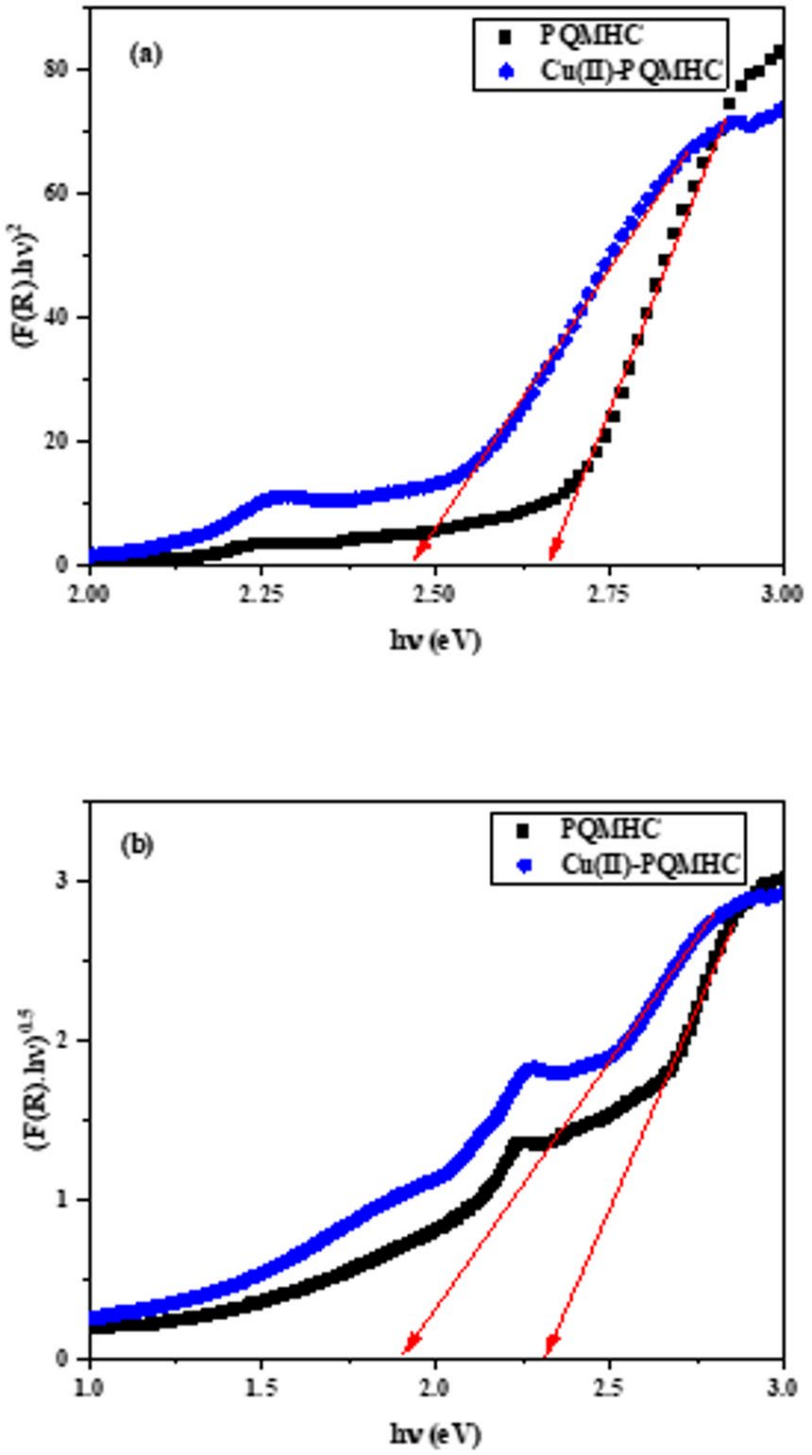
Plot of (a) (F(R) hν)^2^ versus hν and (b) (F(R) hν)^0.5^ versus hν of **PQMHC** ligand and its Copper (II) complex.

Figure 8a and b present the Tauc plots of (F(R) hν)^2^ and (F(R) hν)^0.5^ as functions of photon energy (hν) for the PQMHC ligand and its Cu(II)-PQMHC complex. The plots reveal well-defined linear regions, confirming that the materials exhibit optical transitions of both direct and indirect nature. The optical band gap values extracted from the extrapolation for both direct and indirect allowed transitions as summarized in Table 2. Notably, the Cu(II)–PQMHC complex shows a slight redshift in the absorption edge relative to the free ligand, signifying a narrowing of the band gap upon metal coordination. This redshift may arise from enhanced charge delocalization and orbital overlap between the copper center and the ligand framework.

**Table 2:** Energy gap, refractive index, dielectric constant, and electronegativity for the **PQMHC** ligand and its copper (II) complex for both direct and indirect.

Sample	Transition	Eg (eV)	Method	Moss	Ravindra	Hervé and Vandamme	Reddy	Anani	Kumar and Singh	Hossam, Ibrahim, and Heba	Duffy	Average
**PQMHC**	Direct	2.661	n	2.4444	2.4342	2.4332	2.8618	2.8678	2.4559	2.3237	---	2.5459
			ε	5.9750	5.9252	5.9206	8.1898	8.2243	6.0314	5.3996	---	6.5237
			Δχ^*^	0.8504	0.8592	0.8600	0.5602	0.5569	0.8407	0.9595	0.7153	0.7753
	Indirect	2.305	n	2.5337	2.6549	2.5585	2.9849	2.9390	2.5723	2.4371	---	2.6686
			ε	6.4199	7.0485	6.5459	8.9096	8.6377	6.6165	5.9397	---	7.1597
			Δχ^*^	0.7777	0.6890	0.7587	0.4953	0.5186	0.7484	0.8566	0.6196	0.6830
**Cu(II)-PQMHC**	Direct	2.460	n	2.4929	2.5588	2.5020	2.9281	2.9080	2.5189	2.3854	---	2.6134
			ε	6.2143	6.5475	6.2598	8.5737	8.4565	6.3446	5.6901	---	6.8695
			Δχ^*^	0.8102	0.7585	0.8029	0.5243	0.5349	0.7894	0.9021	0.6612	0.7229
	Indirect	1.896	n	2.6605	2.9085	2.7246	3.1669	3.0208	2.7394	2.5960	---	2.8310
			ε	7.0785	8.4593	7.4236	10.0293	9.1252	7.5044	6.7393	---	8.0514
			Δχ^*^	0.6851	0.5347	0.6426	0.4129	0.4779	0.6332	0.7308	0.5096	0.5784

A comparison of the fitting accuracy for the two transition types indicates that the direct allowed transition provides a more consistent and pronounced linear correlation than the indirect one for both materials. This suggests that electronic transitions in PQMHC and its Cu(II) complex predominantly occur through a direct optical process, characteristic of well-ordered or semi-crystalline molecular structures. The reduction in *E*_g_ upon complex formation enhances the material’s light-harvesting capability, implying potential suitability for optoelectronic and photovoltaic applications.

The refractive index (n) of a material is generally observed to decrease with an increase in the optical energy band gap (E_g_​), suggesting an intrinsic correlation between these two fundamental parameters. Consequently, numerous empirical and semi-empirical models have been proposed to describe this relationship quantitatively. Among the most notable approaches are the Moss relation, Ravindra relation, Hervé and Vandamme relation, Reddy relation, Anani relation, Kumar and Singh relation, and the Hossam, Ibrahim, and Heba relation. Each of these models attempts to establish a predictive framework linking n and Eg​, reflecting the ongoing efforts to better understand and characterize the optical behavior of materials^52,53^.

Moss relationship:

3 $$\:{n}^{4}{E}_{g}=95eV$$

Ravindra relationship:

4 $$\:n=4.084-0.62{E}_{g}$$

Hervé and Vandamme’s relationship:

5 $$\:n=1+{[13.6/({E}_{g}+3.47\left)\right]}^{2}\:$$

Reddy relationship:

6 $$\:{n}^{4}\left({E}_{g}-0.365\right)=154$$

Anani relationship:

7 $$\:{E}_{g}=17-3n\:eV$$

Kumar and Singh’s relationship:

8 $$\:n=3.3668{E}_{g}^{-0.32234}$$

Hossam, Ibrahim, and Heba’s relationship:

9 $$\:n={\left(\left({3.44}^{2}/{E}_{g}^{0.5}\right)-{3.44}^{0.5}\right)}^{0.5}$$

The average refractive index values, calculated using all aforementioned models for both direct and indirect electronic transitions, are summarized in Table 2. Another key parameter influencing the optical and electronic behavior of materials is the dielectric constant (ε), which reflects the material’s ability to store electrical energy in the presence of an electric field. Empirically, the dielectric constant is related to the refractive index (n) by the following expression^44^:

10 $$\:{\epsilon\:}_{\infty\:}={n}^{2}$$

This relationship provides a straightforward means to estimate the dielectric response of a material based on its refractive index. The refractive index values were computed using the aforementioned empirical relations, and the corresponding dielectric constants for the synthesized samples were subsequently determined. These values are presented in Table 2 for both direct and indirect band gap scenarios. The data summarized in Table 2 encompasses the results obtained from all the applied empirical and semi-empirical models used to estimate the refractive indices of the prepared materials.

Optical electronegativity is regarded as a key parameter in characterizing the nature of chemical bonding, and it serves as a basis for estimating various fundamental physical properties. The correlation between the optical band gap and optical electronegativity was extensively explored by Duffy^54^, who sought to describe the metallic or ionic/covalent character of chemical bonds in terms of the electronegativity associated with the forbidden energy gap. In semiconductors and insulators, optical absorption primarily arises from electronic transitions to the conduction band. When an electron is transferred from an anionic to a cationic site, the resulting energy absorption is referred to as charge transfer absorption. Duffy^49^ formalized this concept through the definition of optical electronegativity (Δχ∗), and proposed the following empirical relation:

11 $$\:{{{\Delta\:}{\upchi\:}}^{*}=0.2688E}_{g}$$

More recently, Reddy et al.^55^ proposed an empirical formula that establishes a relationship between the refractive index and the optical electronegativity of solid materials. This approach offers an alternative perspective for estimating the refractive index based on the inherent electronic properties of the material, particularly its optical electronegativity, thereby enhancing the understanding of the material’s optical behavior. The proposed relation is expressed as follows:

12 $$\:{{\Delta\:}{\upchi\:}}^{*}=9.8{e}^{-n}$$

The average values obtained from both the Duffy and Reddy formulas were computed for the cases of direct and indirect electronic transitions. The values indicate that the compounds are covalent^56^.

### Electrochemical properties

To evaluate the electrochemical performance of the synthesized nanomaterials, the surfaces of screen-printed electrodes (SPEs) were modified via drop-casting with the respective nanocomposites. Cyclic voltammetry (CV) and electrochemical impedance spectroscopy (EIS) measurements were then conducted in an electrolyte solution consisting of 0.1 M KCl and 5 mM K₃[Fe(CN)₆]. Ferricyanide/ferrocyanide [Fe(CN)_6_]^3−/4−^ is a well-established and widely used redox couple in electrochemical studies due to its reversible, single-electron transfer mechanism, high solubility, and well-defined redox potential in aqueous media. It serves as a reliable indicator of the electron-transfer kinetics and surface conductivity of modified electrodes.

**Fig. 9:**
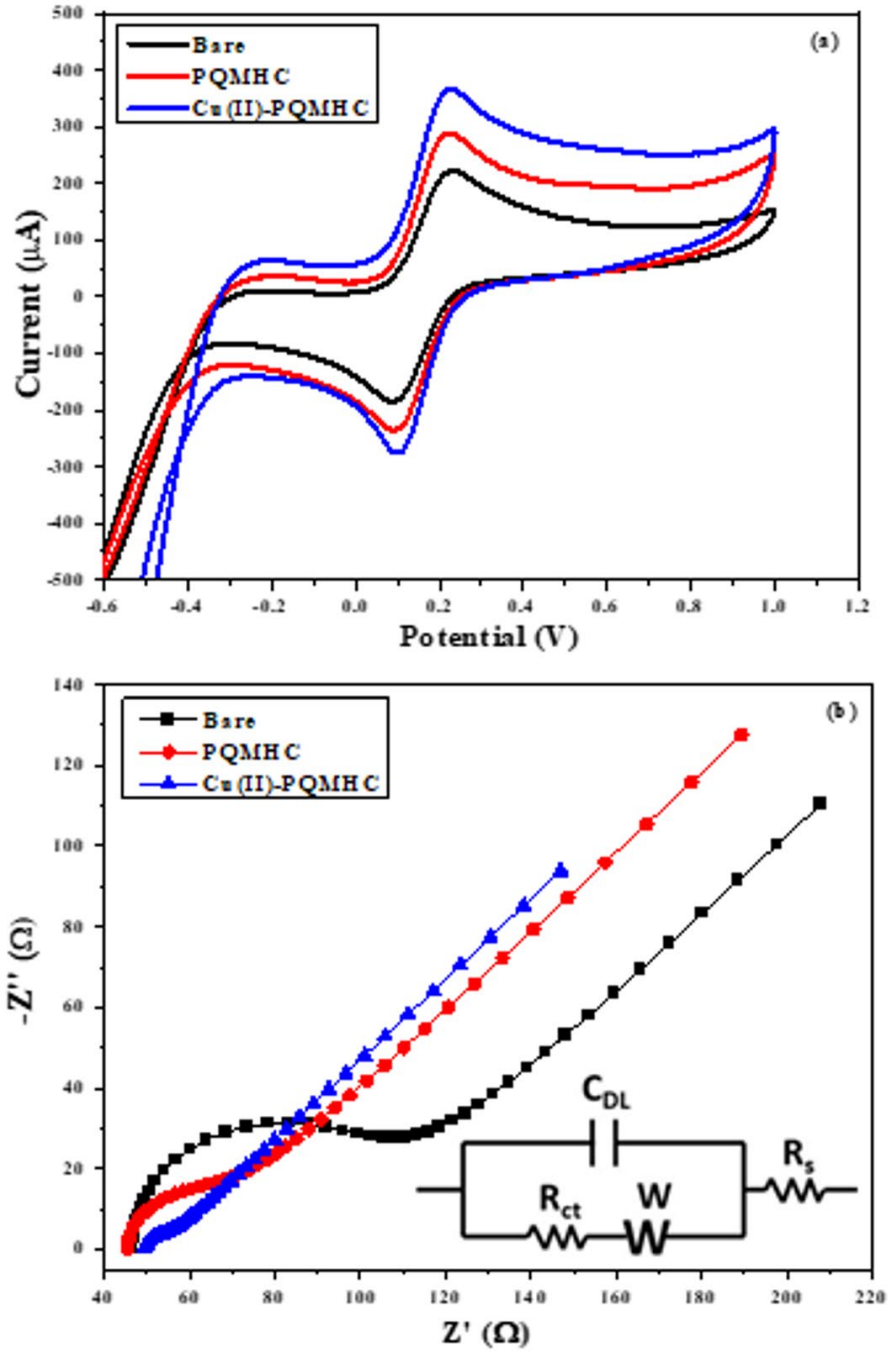
(a) The CV measurements and (b) EIS Nyquist spectra of Bare (unmodified SPE), **PQMHC**, and **Cu(II)-PQMHC** modified SPEs were produced in a solution of ferri / ferrocyanide (5 mM) and 0.1 M KCl. EIS data fitting circuit (inset figure b).

The CV results revealed that all nanomaterial-modified electrodes exhibited significantly higher redox peak currents compared to the unmodified (bare) SPE, indicating enhanced electrochemical activity. As shown in Fig. 9a, the anodic (oxidation) and cathodic (reduction) peak currents increased in the following order:

Bare < PQMHC < Cu(II)-PQMHC, with corresponding oxidation peak currents of 233, 297, and 372 µA, respectively.

Among the tested materials, the Cu(II)-PQMHC**-**modified SPE demonstrated the highest redox current values, confirming its superior electrocatalytic performance. This enhancement is attributed to improved electron transfer at the electrode–electrolyte interface upon complexation.

Electrochemical impedance spectroscopy (EIS) was further employed to assess charge transfer resistance (R_ct_). As depicted in Fig. 9b, the Nyquist plots show a marked decrease in R_ct_ for the modified electrodes. The Cu(II)-PQMHC**-**modified electrode exhibited the lowest charge transfer resistance (R_ct_ = 11.2 Ω), compared to the bare (unmodified electrode) SPE (R_ct_ = 75.6 Ω).

The Nyquist plot shown in Fig. 9b includes an inset depicting the equivalent circuit model used for the quantitative interpretation of the impedance data. The equivalent circuit model used for fitting the EIS data has now been included and illustrated in Fig. 9b. The model follows a Randles circuit configuration, expressed as Rs(Q(RctW)), where Rs represents the solution resistance, Rct the charge-transfer resistance, Q the constant phase element, and W the Warburg diffusion element. In this configuration, Rs is connected in series with a constant phase element (Q), which is parallel to a series combination of Rct and W. This arrangement effectively separates the bulk electrolyte resistance from the interfacial electrochemical processes. The Q(RctW) subcircuit models the Faradaic reaction kinetics and the associated mass-transfer limitations, while the constant phase element accounts for the non-ideal capacitive behavior of the electrode–electrolyte interface.

All fitted parameters derived from this model are summarized in Table 3 and discussed in detail in Sect. 3.4. The results confirm that the Cu(II)-PQMHC complex facilitates the most efficient electron transfer between the redox probe and the electrode surface, demonstrating its superior electrochemical performance.

**Table 3:** The CV and EIS electrochemical characterization parameters obtained for bare (unmodified electrode), and modified electrodes with **PQMHC** ligand and its copper (II) complex.

Electrode modified surface	I_a_ (µA)	I_c_ (µA)	E_oxd_. (V)	E_red_. (V)	E_1/2_ (V)	*R*_s_ (Ω)	*R*_ct_ (Ω)	C µF	W (Ω)
Bare	233	-184	0.23	0.089	0.1595	43	75.6	10.91	0.00257
**PQMHC**	297	-230	0.22	0.089	0.1545	44	46.8	14.52	0.00221
**Cu(II)-PQMHC**	372	-282	0.226	0.099	0.1625	50	11.2	78.88	0.00133

Based on the cyclic voltammetry (CV) and electrochemical impedance spectroscopy (EIS) results, it can be inferred that the Cu(II)–PQMHC complex exhibits promising characteristics, making it a potential candidate for electrochemical applications.

**Fig. 10:**
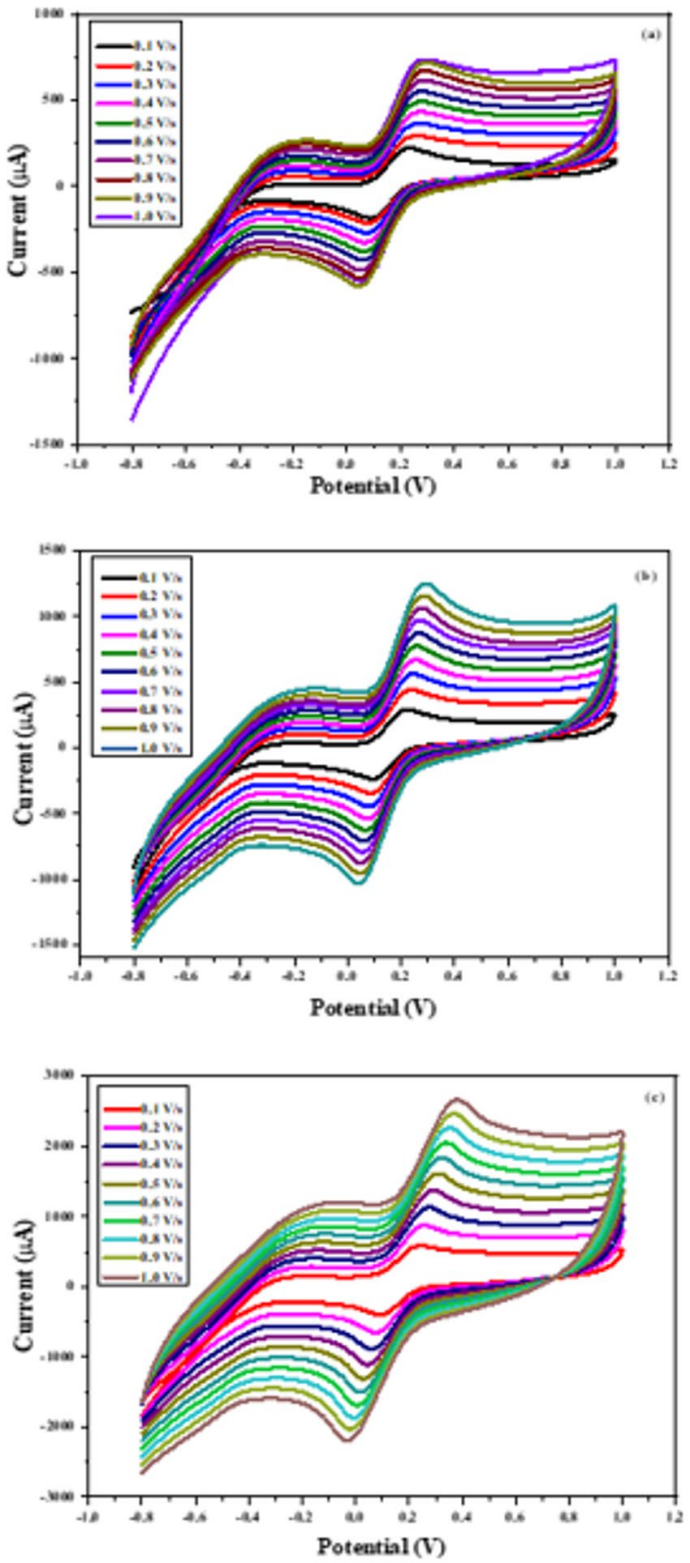
CV measurements at different scan rates for (a) Bare (unmodified SPE), and modified SPEs with a thin film of (b) **PQMHC** and (c) **Cu(II)-PQMHC** complex.

**Fig. 11:**
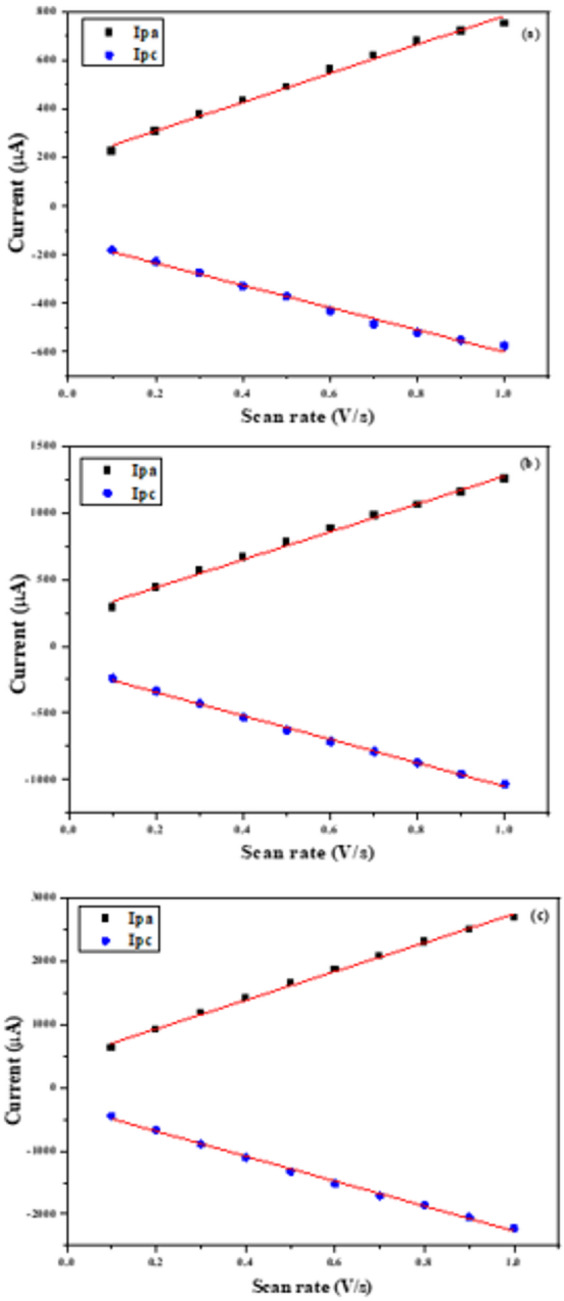
Influence of scan rate changes on the redox peak currents at different scan rates for (a) Bare (unmodified electrode), and modified electrodes with a thin film of (b) **PQMHC** and (c) **Cu(II)-PQMHC** complex.

To further investigate the electrochemical properties of the materials-modified SPEs, voltametric experiments were conducted at varying scan rates in a ferricyanide (FCN) solution. As illustrated in Figs. 10 (a–c), all tested electrodes displayed a linear increase in redox current with increasing scan rate. Notably, the Cu(II)-PQMHC complex-modified electrode (Figs. 11a-c) exhibited the most pronounced linear response, indicating enhanced redox activity.

The effective surface area of the bare and modified electrodes can be determined at 25 °C according to the Randles–Ševčík equation^57^:

13 $$\:{I}_{p}=2.69\times\:{10}^{5}\times\:{n}^{3/2}\times\:A\times\:C\times\:\sqrt{D\times\:\nu\:}$$

Where *Ip* is the peak current (A), *n* the number of electrons transferred, *D* the diffusion coefficient (cm²/s), *A* the electroactive surface area (cm²), *C* the FCN concentration (mol/L), and *ν* the scan rate (V/s).

**Fig. 12:**
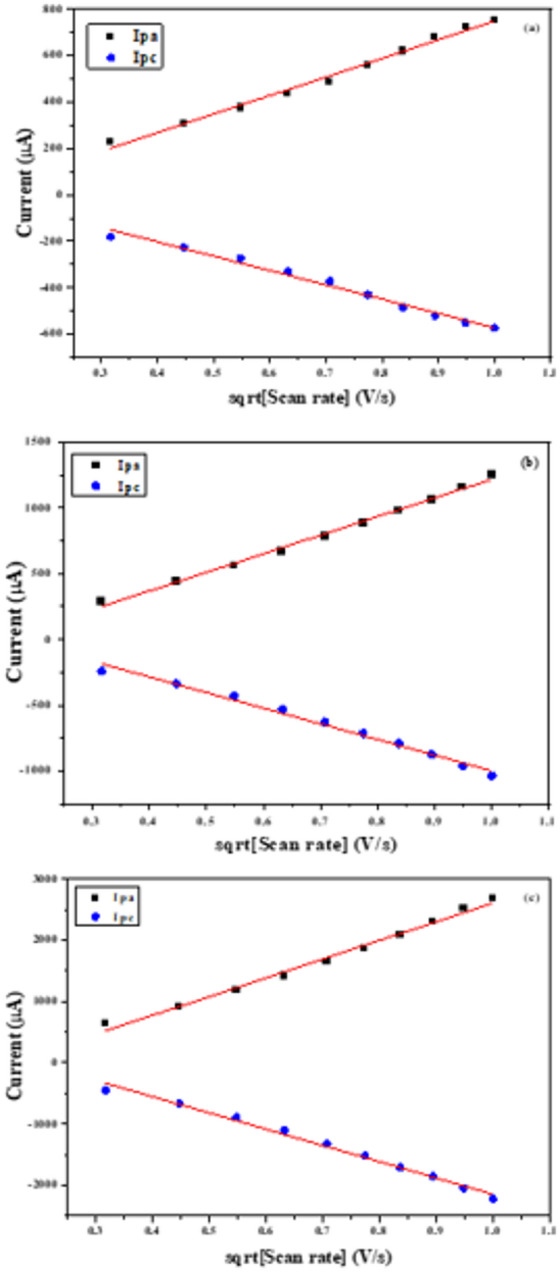
The relationship between the square root of scan rate vs. the redox peak currents for (a) Bare (unmodified electrode), and modified electrodes with a thin film of (b) **PQMHC** and (c) **Cu(II)-PQMHC** complex.

The anodic (Ipa) and cathodic (Ipc) peak currents were plotted against the square root of the scan rate (Figs. 12a-c), yielding a strong linear relationship with a correlation coefficient of 0.9981. Based on this, the effective electrochemically active surface areas were calculated for both unmodified and modified electrodes. The bare SPE showed an active area of 0.16 cm², while modifications with PQMHC and Cu-PQMHC increased the areas to 0.28 cm² and 0.61 cm², respectively. Remarkably, the Cu-PQMHC modification resulted in nearly sixfold enhancement in surface area.

The ECSA values obtained from the Randles–Ševčík analysis are larger than the nominal geometric area of the screen-printed electrode. This apparent discrepancy arises from the nanostructured and porous morphology of the PQMHC and Cu–PQMHC films, which expose a substantially greater electrochemically accessible surface, consistent with similar reports for nanostructured and hybrid-modified carbon electrodes^58–60^.

### Chronoamperometric Estimation of peroxide

The primary aim of this study is to employ novel material with superior electrochemical characteristics and fast, direct electron transfer capabilities for use in sensing applications. Accordingly, the synthesized materials were investigated as modifiers for screen-printed electrodes (SPEs) to fabricate non-enzymatic electrochemical sensors for hydrogen peroxide (H_2_O_2_) detection.

To further optimize sensor performance, the influence of phosphate buffer solution (PBS) pH on the electrochemical oxidation of hydrogen peroxide was evaluated using the Cu(II)-PQMHC modified SPE. The study was conducted using the chronoamperometry (CA) technique.

**Fig. 13:**
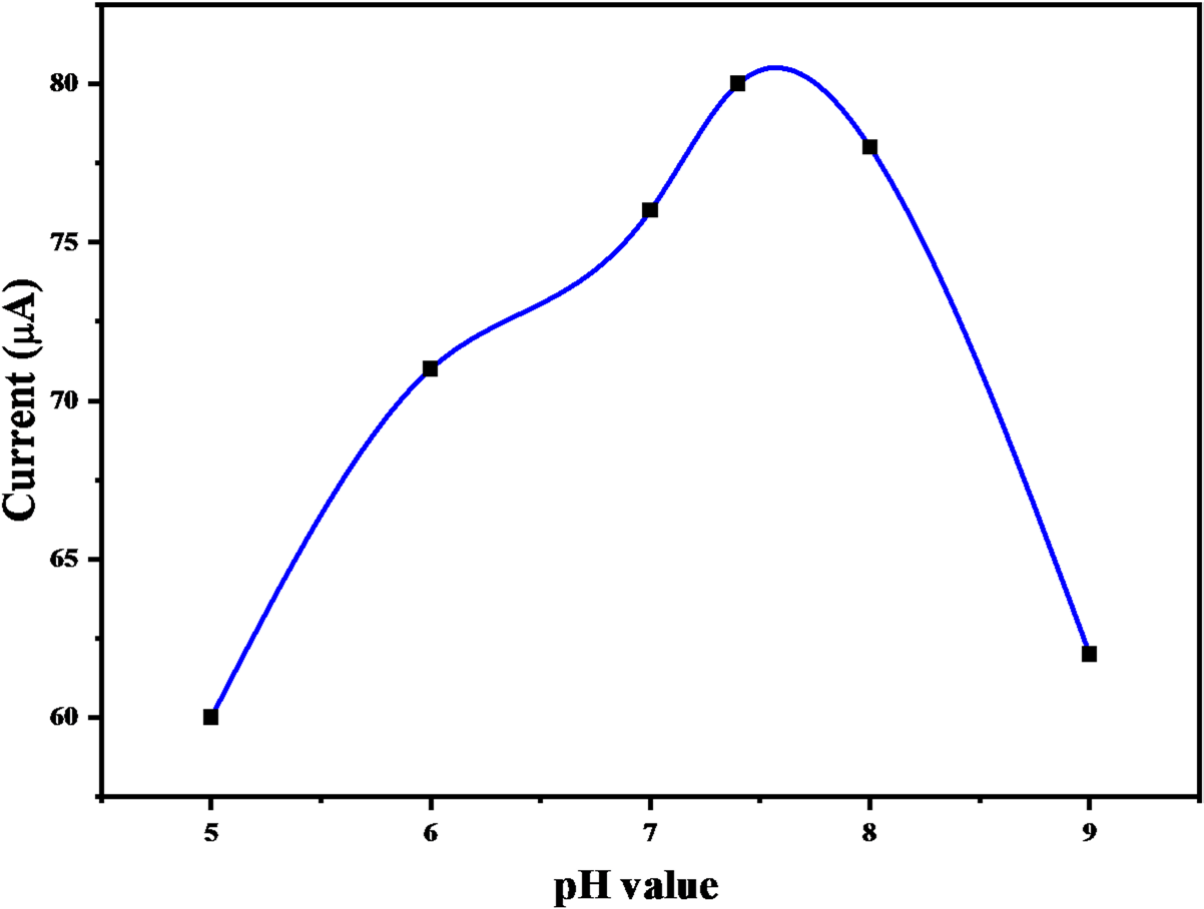
The chronoamperometric response of the modified electrode with a thin film of **Cu(II)-PQMHC** complex in a solution of phosphate buffer containing a fixed concentration of peroxide (400µM) at different pHs.

As illustrated in Fig. 13, the oxidation current of H_2_O_2_ increased progressively with rising pH values, reaching a maximum at pH 7.4. Beyond this point, a gradual decline in peak current was observed, indicating reduced electrocatalytic activity under more alkaline conditions.

The observed trend suggests that neutral pH (7.4) offers the most favorable conditions for efficient electron transfer and catalytic oxidation of H_2_O_2_ at the modified electrode surface. Consequently, PBS at pH 7.4 was selected as the optimal supporting electrolyte for all subsequent experiments.

**Fig. 14:**
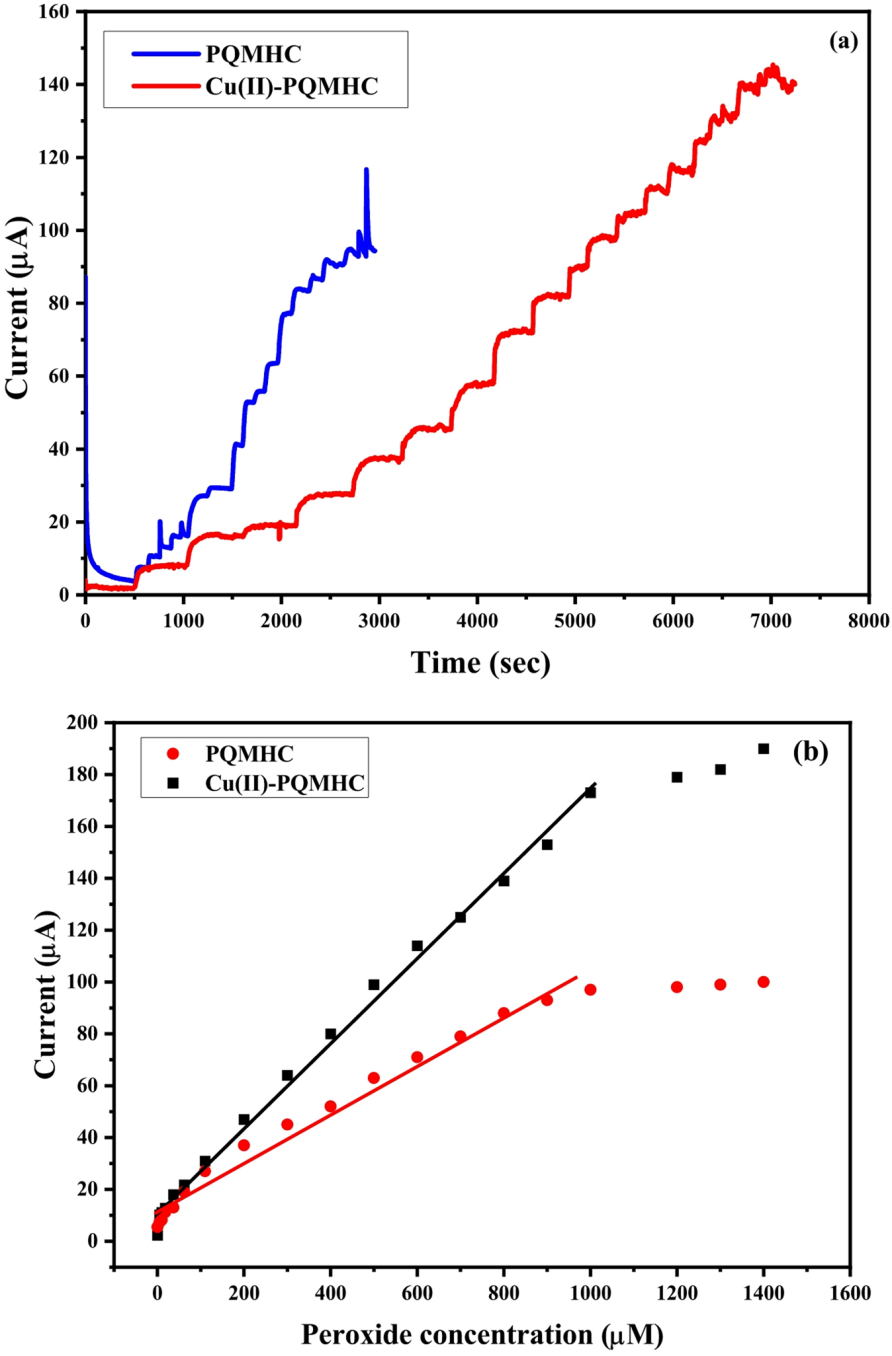
(a) The chronoamperometric response (b), the corresponding curve of calibration between the H_2_O_2_ concentration and the resultant current response, (c) reproducibility test, (d) stability study, and (e) Interference study of modified electrodes with a thin film of **PQMHC** ligand and its Copper (II) complex.

Cyclic voltammetry (CV) confirmed the ability of the Cu(II)-PQMHC modified SPE to directly oxidize hydrogen peroxide (H_2_O_2_), demonstrating the nanocomposite’s effective electrocatalytic properties. Building on these results, chronoamperometry (CA) was employed to further assess the sensor’s analytical performance. Hydrogen peroxide was added at defined concentrations and fixed time intervals, and the corresponding current responses were recorded for each addition, as presented in Fig. 14a.

The calibration curves between hydrogen peroxide concentration and current response (Fig. 14b) also demonstrated high sensitivity and reproducibility. The current response increased proportionally with H_2_O_2_ concentration in the range of 0.05 to 1000 µM. The sensitivity was calculated as 0.163 µA/µM, with an R² value of 0.995. Notably, this method yielded the lowest LOD and LOQ values among the techniques tested: 0.009 µM and 0.01 µM, respectively.

The catalytic mechanism of the peroxide detection performed undergoes a reversible Cu(II)/Cu(I) redox process of Cu(II)-PQMHC complex facilitating electron transfer then hydrogen peroxide interacts with the reduced Cu(I) centers, leading to its catalytic decomposition into water and oxygen via a redox-mediated cycle. The pyranoquinoline semicarbazone ligand enhances electron delocalization through ligand-to-metal charge transfer (LMCT), which stabilizes the Cu redox states and accelerates the electrocatalytic process. A scheme of the proposed mechanism has also been illustrated as Fig. 2.

For reproducibility study, five independent electrodes were fabricated and tested under identical conditions (see Fig. 14c), and the relative standard deviation (RSD) of the current response was found to be below 1.2%. Stability of the proposed sensor retained approximately 98% of its initial response after 2 months of storage at room temperature, indicating good long-term stability (as shown in Fig. 14d). The effect of common interfering substances such as glucose, ascorbic acid, urea and uric acid was examined (see Fig. 14e). The sensor displayed negligible interference, confirming its excellent selectivity for H₂O₂ detection.

These results confirm that the Cu(II)-PQMHC**-**modified SPE exhibits excellent sensitivity, a broad linear detection range, and a low detection limit for H_2_O_2_, making it a highly effective platform for non-enzymatic peroxide sensing. These findings affirm the Cu(II)-PQMHC complex as a promising and efficient material for sensitive and wide-range peroxide detection.

In a comparison between the proposed sensor analytical performance (detection limit, linear range, and sensitivity) with several recent reports on non-enzymatic H₂O₂ sensors (see Table 4). The results clearly show that the Cu(II)-PQMHC sensor exhibits a competitive or superior detection limit and sensitivity compared with other reported systems, highlighting its efficiency and potential practical relevance.

**Table 4:** Comparison between the proposed sensor and other non-enzymatic sensors for hydrogen peroxide detection.

Electrode Applied	potential (V)	Linear range (µM)	Detection limit (µM)	Sensitivity (µA/µM)	References
CNTs/ LFO	0.7	0.1–500	0.005	1.19	^61^
CdO-ZnO-P_2_O_5_ doped with different tungsten (CZWP)	0.7	1.0–1000	0.025	-	^41^
Acrylate polymeric nanocomposites embedded with some transition metals, Ni (II), Fe (III), and Cu (II) triazole complexes	-	1–1000	0.015	-	^29^
CNT-NiCo_2_O_4_	0.7	1–310	0.015	-	^62^
[poly(MMA/DMAEMA/CHAA)]	0.7	1–800	0.0085	-	^63^
[poly(MMA/DMAEMA/AA)] embedded metal oxide (MO)	0.7	1–1000	0.03	-	^64^
BaTi_0.7_Fe_0.3_O_3_@NiFe_2_O_4_ nanocomposites	0.7	0.1–650	0.01		^43^
GO/MnO_2_/CNTs	0.7	1.0–210	0.08	--	^65^
MnO_2_/Cellulose	0.7	0.2–400	0.04	0.70	^66^
Calcium phosphate/ Cu_x_Fe_3−x_O_4_ core-shell nanoceramics	0.7	2.5–200	0.8	0.88	^67^
MnCo_2_O_4_/CNTs	0.7	0.1 − 180	0.1	-	^68^
Pyranoquinoline (PQMHC) / Cu(II) Complex	0.7	0.05–1000	0.009	0.163	This work

## Conclusion

The structural, morphological, and optical characteristics of the PQMHC ligand and its Cu(II) complex were explored in this study. XRD analysis confirmed distinct crystallographic systems-orthorhombic for the ligand and monoclinic for the complex, verifying effective metal coordination. The nanoscale crystallite sizes and compressive strain identified through Williamson–Hall analysis reflect the integrity of the materials’ microstructure. FE-SEM imaging revealed nanofiber morphologies with narrower size distribution in the Cu(II)-complex, suggesting enhanced structural uniformity upon coordination. Optical measurements revealed both direct and indirect electronic transitions. Moreover, empirical models enabled estimation of the refractive index and dielectric constant. The observed nanostructural features and optical behavior highlight the potential of these materials for integration into sensors and other optoelectronic devices. Electrochemical investigations confirmed the superior electrocatalytic activity, efficient electron transfer, and enhanced conductivity of the Cu(II)-PQMHC complex. These properties enabled their excellent performance as non-enzymatic sensors for hydrogen peroxide detection, exhibiting high selectivity, a broad linear detection range, and low detection limits across the chronoamperometry (CA) technique. The enhanced electrochemical response highlights their strong potential for applications in biosensing and environmental monitoring. Overall, the results demonstrate the versatility and effectiveness of these hybrid nanomaterials, supporting their integration into advanced electrochemical sensor platforms and promoting their use in sustainable, next generation sensing technologies.

**Contributions**.

A.A. El-Saady: involved in structure, and optical measurements, formal analysis of XRD, SEM, and optical properties measurements, writing-original draft, and final revision of the manuscript plagiarism. A.A.M. Farag: took part in final revision of all the figures and the manuscript writing. Magdy A. Ibrahim: took part in preparing the samples and final revision of the manuscript. A. M. Mansour: played a role in analyzing and interpreting the data, contributing reagents, materials, analysis tools, or data, and writing the paper. M.M. El-Nahass: took part in the final revision of the manuscript writing. Nesma Salah: took part in preparing the samples and final revision of the manuscript writing. Hend S. Magar: scrutinized and explained the electrochemical data, including materials, analysis tools, and data, and also drafted the paper.

## Data Availability

The datasets used and/or analysed during the current study available from the corresponding author on reasonable request.
